# Causal Genomic and Epigenomic Network Analysis emerges as a New Generation of Genetic Studies of Complex Diseases

**DOI:** 10.4172/2329-9002.1000e113

**Published:** 2015-05-30

**Authors:** Momiao Xiong

**Affiliations:** Human Genetics Center, Department of Biostatistics, The University of Texas School of Public Health, Houston, TX 77030, USA

In the past decade, rapid advances in genomic technologies have dramatically changed the genetic studies of complex diseases. Genome-wide association studies (GWAS) have been widely used in dissecting genetic structure of complex diseases. As of December 18th, 2014, A Catalog of Published Genome-Wide Association Studies (GWAS) had reported significant association of 15,177 SNPs with more than 700 traits in 2,087 publications [1]. However, numerous studies reported that the genetic loci identified by GWAS collectively explain only < 10% of genetic variation across the population in most complex diseases. About 90% of the heritability of common diseases are unexplained by a large number of identified GWA loci. Each variant usually has weak effect and make small and mild contributions to the disease. More than 1,000 loci for many complex diseases have been identified [2]. Although extremely large number of samples are collected and whole genome sequencing studies will be conducted very soon, which will lead to reducing he fraction of missing heritability, a large proportion of heritability will be still missing under the paradigm of single trait genetic analysis. The methods for heritability estimation and single trait genetic study paradigm are questionable.

A biological system consists of multiple phenotypes. The multiple phenotypes are correlated. It has been reported that more than 4.6% of the SNPs and 16.9% of the genes in previous genome-wide association studies (GWAS) were significantly associated with more than one trait [3]. These results demonstrate that genetic pleiotropic effects likely play a crucial role in the molecular basis of correlated phenotype [4]. The heritability of individual phenotype cannot reveal complicated genotype-phenotype structure and is highly unlikely to fully capture the structure of heritability of multiple phenotypes. Furthermore, the estimation of heritability by a single trait approach might be inaccurate. The concept of heritability should be extended from a single trait to multiple traits.

Consider *k* traits. The breeding and phenotype values for *k* traits are denoted by a *k* dimensional vector

*A*= *[A_1,…,_ A_k_]* and *P*= *[P_1_,… P,_k_]^T^*, respectively. A breeding equation is given by

(1) A=HP

Where *H* is a heritability matrix and denoted by

$$H=[\begin{array}{ccc} h_{1} & \cdots & h_{1k}\\ \vdots & \ddots & \vdots \\ h_{k1} & \cdots & h_{k} \end{array}]$$

Suppose that the phenotype can) be decomposed as a summation of additive effect, dominant effect and environment effect:k

(2) P=A+D+E,where

A, D and E represent the genetic additive, dominant and environmental effect, respectively. Denote the covariance matrix between the breeding value and phenotype values by

$$\text{cov}(A,P)=[\begin{array}{ccc} \text{cov}(A_{1},P_{1}) & \cdots & \text{cov}(A_{1},P_{k})\\ \vdots & \ddots & \vdots \\ \text{cov}(A_{k},P_{1}) & \vdots & \text{cov}(A_{k},P_{k}) \end{array}]$$

and variance-covariance matrix of the phenotype *P* by

$$\text{var}(P)=[\begin{array}{ccc} \text{var}(P_{1}) & \cdots & \text{cov}(P_{1},P_{k})\\ \vdots & \ddots & \vdots \\ \text{cov}(P_{k},P_{1}) & \cdots & \mathrm{var}(P_{k}) \end{array}]$$

It is known that

$$\text{cov}(A_{i},P_{j})=\text{cov}(A_{i},A_{j})+\text{cov}(A_{i},D_{j})+\text{cov}(A_{i},E_{j}),$$

which implies that

$$\text{cov}(A,P)=[\begin{array}{ccc} \text{cov}(A_{1},A_{1})+\text{cov}(A_{1},D_{1})+\text{cov}(A_{1},E_{1}) & \cdots & \text{cov}(A_{1},A_{k})+\text{cov}(A_{1},D_{k})+\text{cov}(A_{1},E_{k})\\ \vdots & \ddots & \vdots \\ \text{cov}(A_{k},A_{1})+\text{cov}(A_{k},D_{1})+\text{cov}(A_{k},E_{1}) & \vdots & \text{cov}(A_{k},A_{k})+\text{cov}(A_{k},D_{k})+\text{cov}(A_{k},E_{k}) \end{array}]$$

It follows from equation (1) that the heritability matrix is estimated by

(3) $$H=\text{COV}(A,P){\left[\text{var}(P)\right]}^{-1}$$

Equation (3) shows that the heritability of the *i^th^* trait *h_ii_* is a function of the genetic covariance between the *i^th^* trait and other traits. In other words, the heritability of each trait is influenced by its correlation with other multiple traits. This clearly demonstrates that the trait by trait genetic study will overlook the influence of other traits. The missing heritability may be due to trait by trait genetic analysis. The joint genetic analysis of multiple traits may increase the heritability.

There has been increasingly consensus that individual genetic and epigenetic variants, individual genes, individual linear pathway and individual trait analysis cannot capture the intrinsic genetic and epigenetic complexity of multiple phenotypes.

To completely capture the heritability, the right research direction is to jointly investigate genetic, expression, miRNA, epigenetic, metabolic variants, physiological traits, medical imaging measurements and environments in multiple traits which are often interactively organized networks. Integrative analysis of genetic, epigenetic, imaging and environmental variation in multiple phenotypes will fully uncover the heritability and facilitate the understanding the mechanism of the complex diseases. The popular methods for integrative analysis are mainly based on correlation and association analysis. These methods cannot efficiently detect, distinguish and characterize the true biological, mediated and spurious pleiotropic effects. Therefore, these approaches may not provide clear biologically or clinical relevant information that allows the mechanisms of genetic effects to be discovered and understood. To overcome these limitations, developing a new framework and novel statistical methods for inferring causal networks of genotype-phenotypes with NGS data and detecting, distinguishing and characterizing the true biological pleiotropic, mediated pleiotropic and spurious pleiotropic effects of genetic variants are urgently needed.

An essential issue for using causal graphs to study genetics of multiple phenotypes is how to accurately and efficiently estimate the structure of causal graph from observational data. Structure learning of casual graphs has been shown to be NP-hard. Early methods for structure learning mainly focused on approximation algorithms, but such methods are unable to ensure the generation of the true causal graph. To obtain the causal graph from observation data as close to the biological causal graph as possible, “score and search”-based methods for exact learning causal graphs of genotype-phenotype to find the best-scoring structures for a given dataset are being developed. The accurate and robust estimation of the genotype-phenotype causal networks by the “score and search” methods will shift the paradigm of genetic studies of correlated multiple phenotypes from association analysis to causal inference, and dramatically facilitate discovery of the mechanism underlying multiple traits.

Although their application to genome-wide genotype-phenotype network construction is difficult due to computational limitations, the “score and search” based causal inference methods are suitable to the phenome-wide association studies where starting phenomics, defined as the unbiased study of a large number of phenotypes in a population. We study the complex networks between multiple expressed phenotypes and genetic variants. Since the number of genetic variants in the phenome-wide association is quite limited and hence the size of the genotype-phenotype network is limited, the required computational time of construction of genotype-phenotype networks using causal inference is in the range the current computer system can reach. Advances in biosensors and sequencing technologies generate large amounts of phenotype and genetic data. Causal genetic and epigenetic network analysis may emerge as a new paradigm of genetic studies of complex traits. The main purpose of this editorial is to stimulate discussion about what are the optimal strategies to facilitate the development of a new generation of genetic analysis. I hope that more and more real data analysis in the future will greatly increase the confidence in causal inference for genotype-phenotype studies.
